# Urinary oxygen tension measurement using a 3-way silicone urinary catheter with enhanced capability for urine collection

**DOI:** 10.1007/s00540-025-03467-0

**Published:** 2025-02-20

**Authors:** Takao Kato, Riku Kobashi, Fumikazu Watanabe, Kaoru Koyama

**Affiliations:** 1 Department of Anesthesiology, Saitama Medical Center, Saitama Medical University, 1981 Kamoda, Kawagoe, Saitama 350-8550 Japan; 2 Fuji Systems Corporation, 472 Akiba, Totsuka, Yokohama, Kanagawa 245-0052 Japan

**Keywords:** Urinary oxygen tension, Acute kidney injury, 3-way urinary catheter, Direct urine correction, Silicone, Oxygen permeability

## Abstract

Acute kidney injury (AKI), particularly during cardiac surgery, severely affects patient outcomes, yet current diagnostics using serum creatinine levels delay detection. This study investigates real-time urinary oxygen tension (PuO_2_) as an alternative early marker and correlates it with renal medullary oxygen tension for potential AKI prediction. We developed a modified 3-way silicone urinary catheter designed for more reliable urine collection and evaluated its performance in terms of PuO_2_ measurement accuracy. The results of the experiment showed that the improved catheter enabled continuous urine collection, but it was suggested that the oxygen permeability of the silicone material may have caused an increase in PuO_2_ level. These results suggest that material selection, particularly oxygen permeability, has a significant impact on PuO_2_ measurements and highlight the need for alternative low-permeability materials to improve the reliability of PuO_2_-based AKI diagnostics.

**Supplementary Information:**

The online version contains supplementary material available at 10.1007/s00540-025-03467-0.

Acute kidney injury (AKI) during the perioperative period, particularly in cardiac surgery, significantly worsens patient outcomes. However, the current diagnostic criterion relies on serum creatinine levels, which peak approximately 72 h after the initial injury, leading to delayed treatment [1]. It has been suggested that renal medullary tissue oxygen tension and bladder urinary oxygen tension (PuO_2_) are correlated in real-time and may aid in early diagnosis of AKI [2]. We have developed a method for intermittent PuO_2_ measurement using a blood gas analyzer. In a preliminary study, our findings indicated that a PuO_2_ level below 130 mmHg at 6 h post-ICU admission and below 88.6 mmHg at 12 h post-ICU admission are predictors of AKI, allowing for earlier diagnosis compared to serum creatinine measurements [3]. However, since the urine collection port is located distally on the urinary catheter, exposure of urine to air resulted in elevated PuO_2_ levels, which was a significant problem.

We also attempted to measure PuO_2_ directly from the bladder using an existing 3-way urinary catheter with a 2-hole distal flush lumen (Silicone Foley Catheter 3-way type, Fuji Systems, Tokyo, Japan), but were successful in only 1 out of 6 cases [4]. To address this issue, we modified a 3-way urinary catheter with a bladder flush lumen penetrating the urine drainage lumen of the catheter to allow for reliable urine aspiration, and occluding the distal hole of the flush lumen (Fig. 1). Here, we report an in vitro study that had the objectives of comparing the urine aspiration performance of unmodified and modified 3-way catheters and confirming the accuracy of PuO_2_ in aspiration from the modified 3-way catheter.

**Fig. 1:**
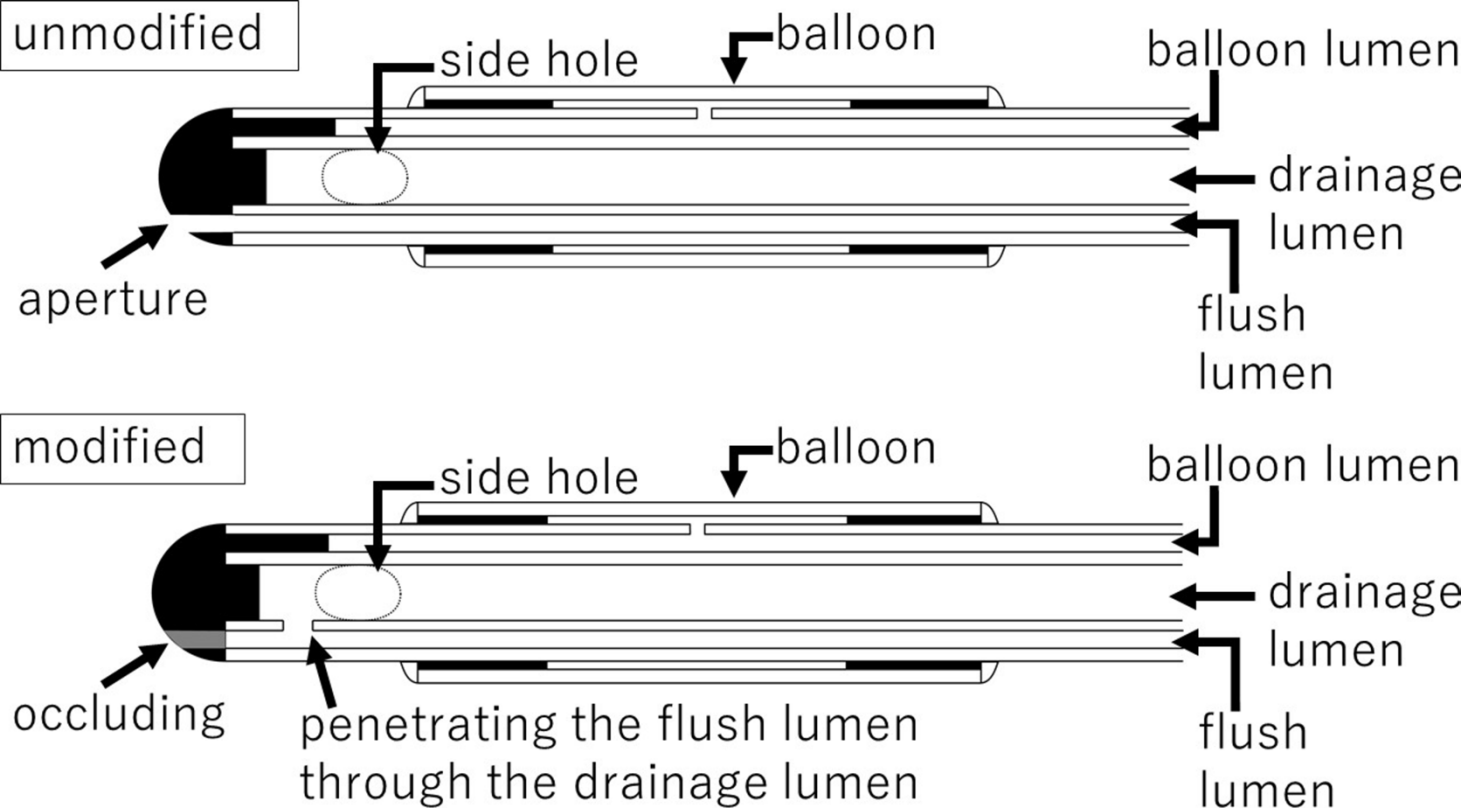
Methods for improving the efficiency of urine collection from the bladder flush lumen of a 3-way urethral catheter. Normally, urine is drained through the side hole and out of the drainage lumen. By occluding the tip of the flush lumen and penetrating into the drainage lumen, it is possible to collect urine reliably from the flush lumen

First, to confirm that urine can be collected, an experimental system was created in which a high-density polyethylene bag was used as the bladder wall and a funnel as the outflow channel for urine from the bladder. Each of 3-way catheter was inserted into this system to compare the performance for urine aspiration before and after the modifications. Second, purified water with an oxygen partial pressure of 65 mmHg or less was placed in a beaker, and the tip of the modified 3-way catheter was immersed in the water. For anaerobic and more stable urine aspiration, suction was performed from the bottom of a glass beaker covered the top with a 1 ml syringe (the outer cylinder is made of polypropylene, and the gasket is made of thermoplastic elastomer). The results were compared with PuO_2_ suctioned directly from purified water using a syringe (*n* = 3). A RAPID Lab™ 348EX (Siemens Diagnostics, Munich, Germany) blood gas analyzer was used for all measurements, and the measurement was taken within seconds of urine collection. Finally, the same experiment was performed using a polyvinyl chloride (PVC)-coated catheter to account for the effects of oxygenation from the urethral catheter portion exposed to the outside air (Supplementary Fig. S1).

The unmodified 3-way catheter could not sustain aspiration due to both distal holes being occluded by the vacuum pressure. In contrast, the modified 3-way catheter allowed for continuous aspiration. However, PuO_2_ with aspiration through the infusion lumen of the modified catheter was 27–55 mmHg higher than that with direct aspiration from the purified water (Table 1). PuO_2_ was 15–27 mmHg higher when aspirated from a PVC-coated urinary catheter than when aspirated directly (Supplementary Fig. S2).

**Table 1:** Comparison of PuO_2_ between direct fluid aspiration and fluid aspiration through a modified urinary catheter. The fluid was adjusted to PuO_2_ of 65 mmHg or less

PuO_2_ (mmHg)	Direct fluid aspiration	Fluid aspiration through a modified urinary catheter
First measurement	56.4	111.4
Second measurement	60.1	87.8
Third measurement	59.9	92.7
Mean ± standard deviation	58.8 ± 2.1	97.3 ± 12.5

These results show that continuous aspiration is feasible after occluding the distal holes and enabling urine aspiration from the tip of the catheter. However, during the aspiration process, significant PuO_2_ elevation was observed due to oxygenation. Silicone is known for its high oxygen permeability, and probably caused substantial oxygenation of the urine as it passed through the flush lumen, leading to the increased PuO_2_ measurement. In the last experiment, it is believed that the increase in PuO_2_ was reduced because the PVC coating of the urethral catheter reduced the oxygen transfer from the silicone membrane. However, the increase in PuO_2_ could not be completely controlled. It is also reasonable to think that anaerobic urine collection was not possible in our previous study [4]. Latex, although not as oxygen-permeable as silicone, also has considerable oxygen permeability, suggesting that similar oxygenation effects might occur [5].

The reported values of PuO_2_ vary widely, making it challenging to establish a standard reference range [6], and this variability is likely to be influenced by the high oxygen permeability of silicon. Thus, when interpreting PuO_2_ measurements, it is important to consider the oxygen permeability of the materials in the urine outflow path up to the measurement site. There is a need to explore the use of materials with low oxygen permeability for constructing the outflow path (including not only the urinary catheter but also the syringe used to collect urine) and to investigate the effects of these materials.

## Supplementary Information

Below is the link to the electronic supplementary material.

Supplementary file1 (JPG 109 KB)

Supplementary file2 (PPTX 44 KB)

## Data Availability

All data generated or analyzed during this study are included in this published article.
