# Supplementary figures and images for: Urinary oxygen tension measurement using a 3-way silicone urinary catheter with enhanced capability for urine collection
Source: J Anesth. 2025 Feb 20;39(2):318–20. doi: 10.1007/s00540-025-03467-0 (PMC11937057; doi:10.1007/s00540-025-03467-0)

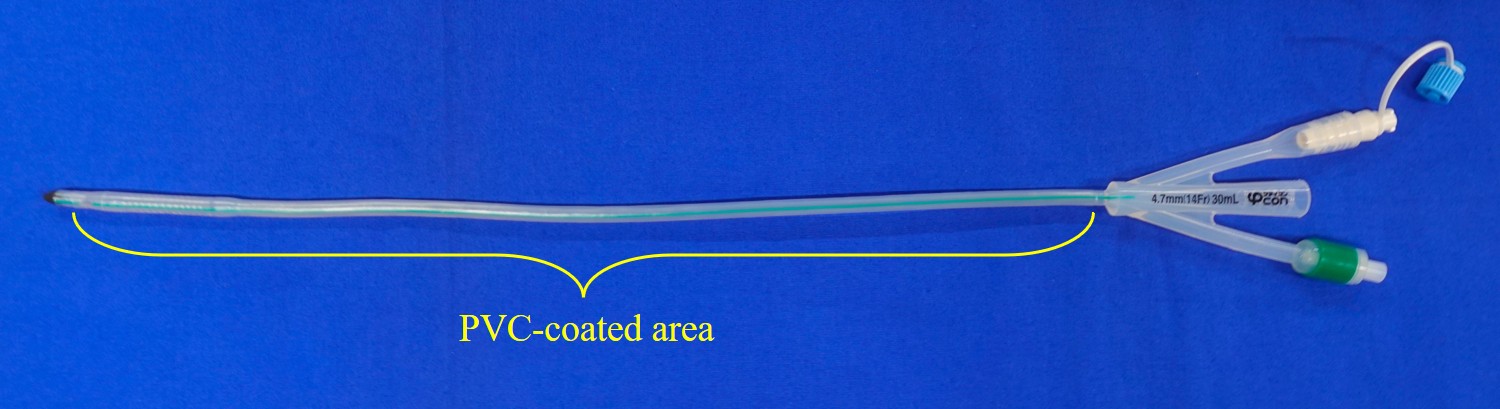

Supplement: Supplementary file 1 — Supplementary file1 (JPG 109 KB) [file 540_2025_3467_MOESM1_ESM.jpg]
