# Supplementary material for: Urinary oxygen tension measurement using a 3-way silicone urinary catheter with enhanced capability for urine collection
Source: J Anesth. 2025 Feb 20;39(2):318–20. doi: 10.1007/s00540-025-03467-0 (PMC11937057; doi:10.1007/s00540-025-03467-0)
Supplement: Supplementary file 2 — Supplementary file2 (PPTX 44 KB) [file 540_2025_3467_MOESM2_ESM.pptx]

## Slide 1
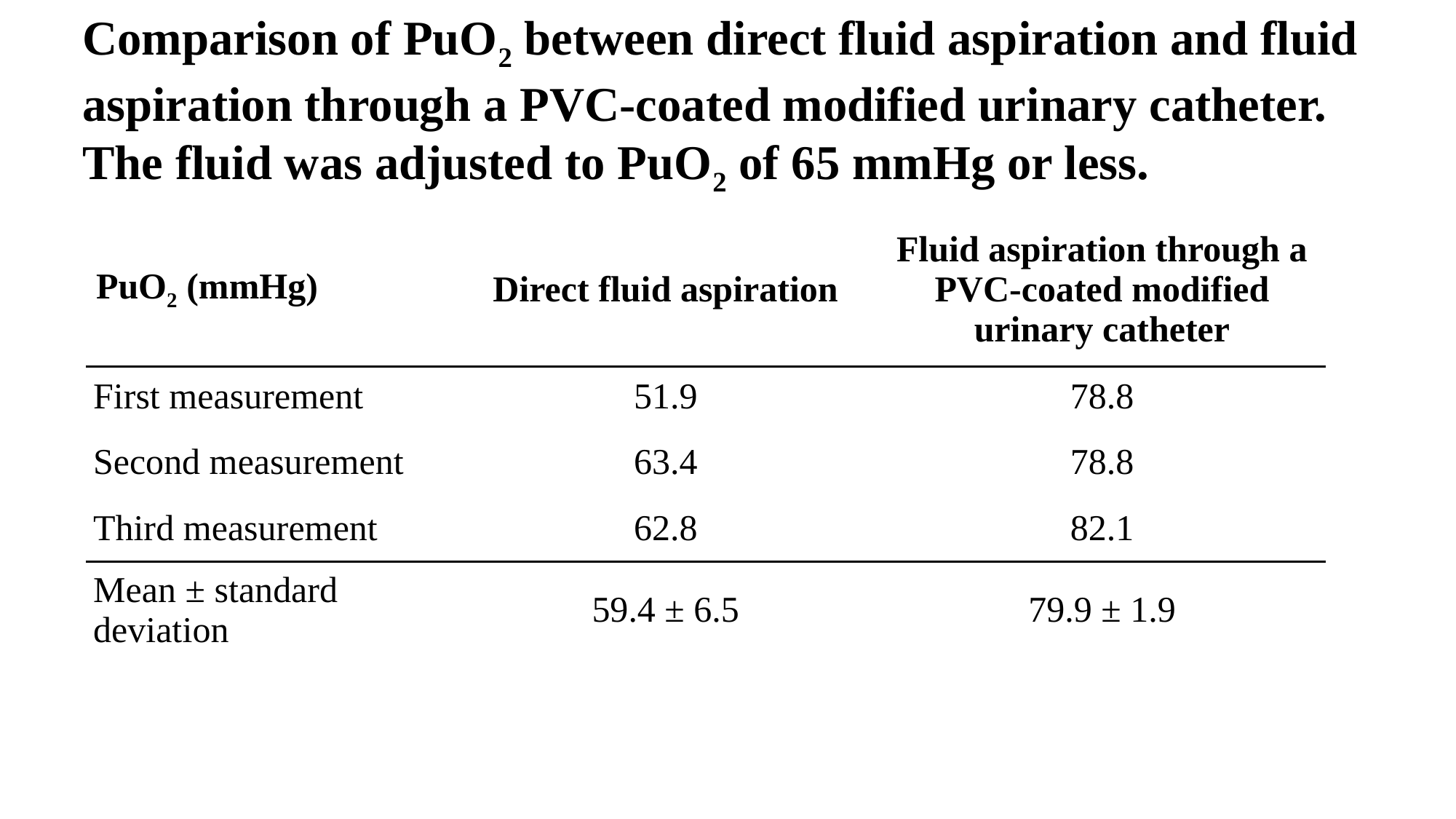

Comparison of PuO2 between direct fluid aspiration and fluid aspiration through a PVC-coated modified urinary catheter.
The fluid was adjusted to PuO2 of 65 mmHg or less.
| PuO2 (mmHg) | Direct fluid aspiration | Fluid aspiration through a PVC-coated modified urinary catheter |
| --- | --- | --- |
| First measurement | 51.9 | 78.8 |
| Second measurement | 63.4 | 78.8 |
| Third measurement | 62.8 | 82.1 |
| Mean ± standard deviation | 59.4 ± 6.5 | 79.9 ± 1.9 |
